# Single-cell RNA-seq reveals the effects of the *FecB* mutation on the transcriptome profile in ovine cumulus cells

**DOI:** 10.1038/s41598-024-64001-y

**Published:** 2024-06-07

**Authors:** Xiaofei Guo, Yi Fang, Rong Liang, Xiangyu Wang, Jinlong Zhang, Chunxiao Dong, Biao Wang, Yu Liu, Mingxing Chu, Xiaoshen Zhang, Rongzhen Zhong

**Affiliations:** 1 Jilin Province Feed Processing and Ruminant Precision Breeding Cross Regional Cooperation Technology Innovation Center, Jilin Provincial Laboratory of Grassland Farming, State Key Laboratory of Black Soils Conservation and Utilization, Northeast Institute of Geography and Agroecology, Chinese Academy of Sciences, Changchun, 130102 China; 2 Tianjin Key Laboratory of Animal Molecular Breeding and Biotechnology, Tianjin Engineering Research Center of Animal Healthy Farming, Institute of Animal Science and Veterinary, Tianjin Academy of Agricultural Sciences, Tianjin, 300381 China; 3 State Key Laboratory of Animal Biotech Breeding, Institute of Animal Science, Chinese Academy of Agricultural Sciences (CAAS), Beijing, 100193 China; 4 Key Laboratory of Animal Production, Product Quality and Security, Ministry of Education, Jilin Agricultural University, Changchun, 130118 China

**Keywords:** Single-cell RNA-seq, Cumulus cells, Follicle, *FecB*, Sheep, Animal breeding, Gene expression, Genetic markers, Mutation

## Abstract

Genetic variations in the ovine ovulation rate, which are associated with the *FecB* mutation, provide useful models by which to explore the mechanisms regulating the development of mammalian antral follicles. In order to study the effects of the *FecB* mutation on cumulus cell differentiation, preovulatory follicles were aspirated and cumulus cells were isolated from three *FecB* genotypes (homozygous, heterozygous and wild type) of Small Tail Han (STH) sheep superstimulated with FSH. Transcriptome information from tens of thousands of cumulus cells was determined with the 10 × Genomics single-cell RNA-seq technology. Under the superovulation treatment, the observed number of preovulatory follicles in the ovaries of *FecB* carriers was still significantly higher than that in the wild-type (*P* < 0.05). The expression patterns of cumulus cells differed between *FecB* carriers and wild-type ewes. The screened cumulus cells could also be further divided into different cell clusters, and the differentiation states and fates of each group of cumulus cells also remained different, which supports the notion that heterogeneity in gene expression is prevalent in single cells. The oxidative phosphorylation pathway was significantly enriched in differentially expressed genes among the cell differentiation branch nodes of cumulus cells and among the differentially expressed genes of cumulus cells from the three genotypes. Combined with the important role of oxidative phosphorylation in the maturation of COCs, we suggest that the oxidative phosphorylation pathway of cumulus cells plays a crucial role in the differentiation process of cumulus cells and the mutation effect of the *FecB* gene.

## Introduction

Genetic variations in the ovine ovulation rate, which are associated with the *FecB* mutation, provide useful models by which to explore the mechanisms regulating the development of antral follicles^1^. Improving the litter size is also an important goal in meat sheep^2^. As early as the 1980s, Davis et al. proposed the hypothesis that the majority of Booroola sheep carry a gene(s) with a major effect on the ovulation rate^3^. This gene appeared to be additive for the ovulation rate and partially dominant for the litter size^3,4^. As this high fecundity trait was present in the Booroola breed, the gene was termed the Booroola fecundity (*FecB*) gene^5^. The *FecB* mutation affects the Bone morphogenetic protein receptor type-1B (*BMPR1B*) gene on sheep autosome 6; due to the A746G mutation in the coding region of the *BMPRIB* gene, the amino acid substitution of Q249R was triggered in its protein sequence^1,6,7^. Marker-assisted selection (MAS) with this gene can be used to quickly generate high-fecundity sheep^8^.

The molecular mechanisms of the *FecB* mutation regarding the ovulation rate or litter size, and the effects of this mutation on other reproductive traits, are of considerable interest. There have been many studies of the reproductive phenotypes, the gene expression in the gonadal tissues and the concentrations of small molecules in the various body fluids of *FecB* sheep^9–15^. Wang et al. reported that the estrus performance of *FecB* heterozygote ewes differed from that of *FecB* homozygote and wild-type ewes, and they speculated that the developing ovarian follicles of *FecB* carriers can respond earlier to FSH than those of wild-type ewes^13^. In the ovaries, it is mainly granulosa cells (including cumulus cells) that express the FSHR protein, enabling follicular development in response to FSH^16^. During antrum formation, granulosa cells differentiate into mural granulosa and cumulus cells. Cumulus cells have an intraovarian paracrine communication system that extends from the oocyte to the theca cells^17,18^, enabling signal transmission within the ovary to regulate oocyte growth and maturation^19^.

Studying the gene expression of cumulus cells from ewes with disparate *FecB* genotypes may help to reveal the molecular mechanisms underlying the differences in their ovulation rates. We also put forward the hypothesis that cumulus cells’ expression patterns should differ between *FecB* gene carriers and non-carriers. With the emergence of real-time PCR, microarrays, whole-transcriptome high-throughput sequencing, and Smart-Seq2 single-cell transcription sequencing and other technologies, researchers have been provided with increasingly effective research tools by which to analyze the role of cumulus cells in follicle development, ovulation and reproductive disease diagnosis^20–22^. A droplet-based single-cell RNA-seq technology that enables the encapsulation of tens of thousands of single cells within minutes has been developed^23^, and the 10 × Genomics platform is being advanced, especially in cellular heterogeneity analysis. In the present study, we isolated cumulus cells by aspirating the preovulatory follicles of three *FecB* genotypes (homozygous, heterozygous and wild type) of Small Tail Han (STH) sheep. Using the 10 × Genomics single-cell RNA-seq technology, we aimed at the determination of transcriptome information from tens of thousands of cumulus cells, to explore the heterogeneity in the cumulus cell populations and elucidate the effects of the *FecB* mutation on cumulus cell differentiation. The findings may provide new insights to explain the mechanism behind the high fecundity performance in some ewes and uncover cell markers for cumulus cells.

## Materials and methods

### Animals and grouping

Blood samples were collected by jugular venipuncture into anticoagulation tubes from pluriparous Small Tail Han (STH) ewes aged 3 years old. DNA was extracted and *FecB*-TaqMan probes (Probe-A: AAATATCAGACGGTGTTG-MGB; Probe-G: AAATATCGGACGGTGTTG-MGB) were used for real-time PCR amplification to identify the *FecB* mutation. The real-time PCR procedure for the TaqMan assay was as follows: incubation at 95 °C for 10 min, followed by 40 cycles at 95 °C for 30 s and 60 °C for 60 s. Based on the *FecB* genotyping, five ewes from each of the three *FecB* genotypes (BB, WB and WW) were selected. All experimental procedures were approved by the Science Research Department of the Chinese Academy of Science Animal Care and Use Committee (approval 2022-147-106), and all methods were performed in accordance with the relevant guidelines and regulations. This study was conducted in accordance with the ARRIVE guidelines (https://arriveguidelines.org).

### Experimental treatment and reproductive phenotyping

All of the selected experimental STH ewes with the three *FecB* genotypes were subjected to estrus synchronization and superovulation in April. The specific procedure was as follows: the intramuscular injection of 5 ml vitamin AD (Xixiang Changjiang Animal Drug Co., Hanzhong, Shaanxi, China) was performed, and intravaginal controlled progesterone release device (CIDR; Pharmacia and Upjohn Co., Hartwell, Australia) was inserted into the vagina of each experimental sheep on Day 0; a total dose of 300 IU follicle-stimulating hormone (FSH, Sansheng, Ningbo, China) was injected intramuscularly every 12 h from Day 10 to Day 14, and the CIDR was removed on Day 12. A total dose of 360 IU pregnant mare serum gonadotropin (PMSG, Sansheng, Ningbo, China) was injected intramuscularly 8 h after the CIDR was removed.

Next, 24 experimental STH ewes with the three *FecB* genotypes were selected for reproductive phenotyping. After the removal of the CIDRs, rams with a high serving capacity were selected as teasers, wearing aprons around their hypogastria for the detection of the estrus phenotype, and teasing was performed four times per day (each time with a 6 h interval) until the end of the estrus. Then, the phenotypes of the first estrus and last estrus and the estrus duration for each ewes were analyzed. The number of preovulatory follicles was determined using a laparoscopy procedure at 45 h after CIDR removal. Meanwhile, 5 ewes from each *FecB* genotype group were subjected to the collection of 10 ml jugular blood using BD SST Tubes (BD, San Diego, CA, USA) at 45 h after CIDR removal for the production of serum. The concentrations of follicle-stimulating hormone (FSH), luteinizing hormone (LH), progesterone (P_4_) and estradiol (E_2_) in the serum of each of the selected experimental ewes were detected using radioimmunoassay (RIA) kits, all from the Beijing North Institute of Biological Technology (Beijing, China). The sensitivity and coefficient of variation (CV) for each RIA kit were reported in our previous research^13^.

### Cumulus cell collection

**Figure 1:**
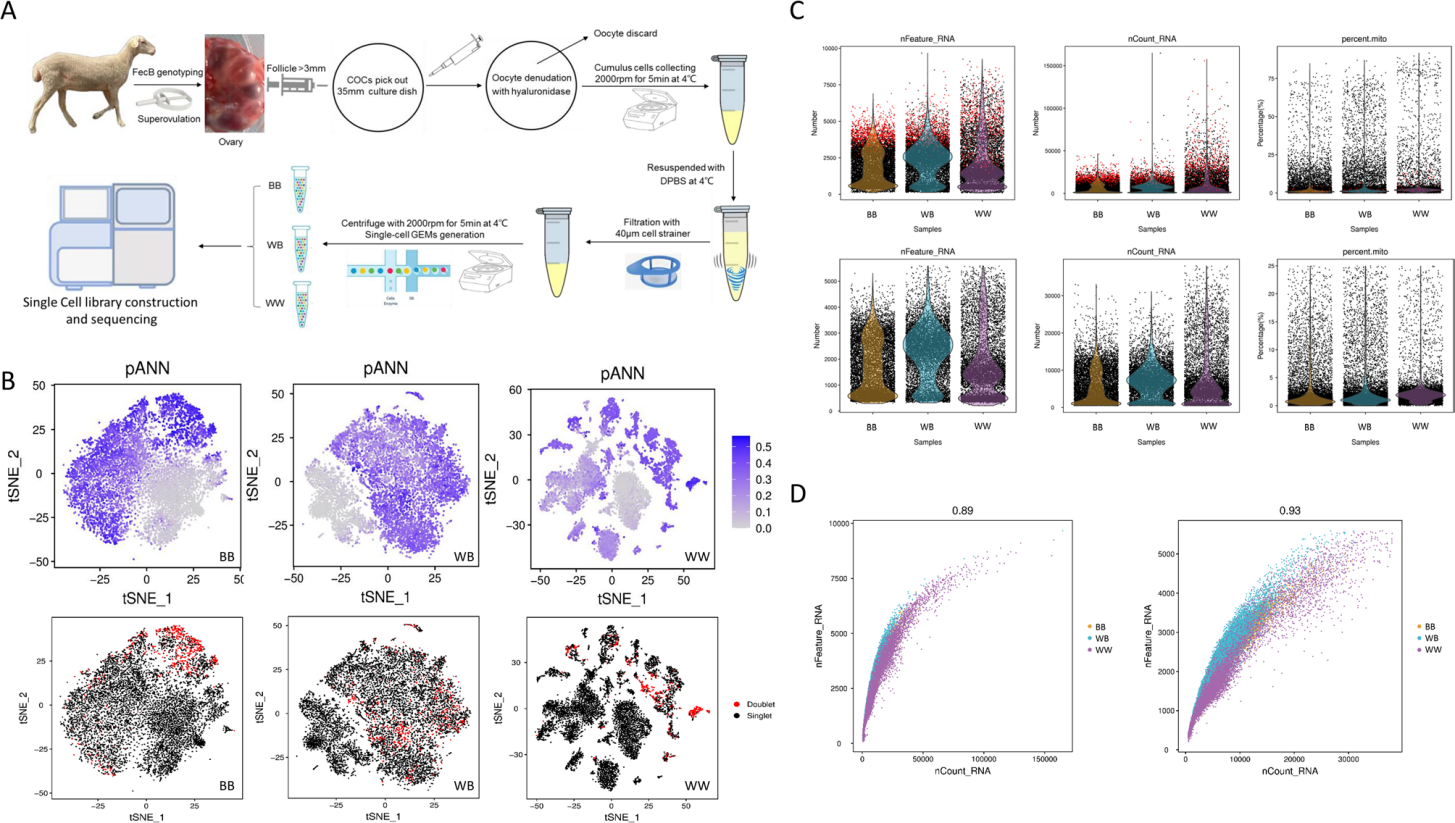
Collection of cumulus cells for Single-Cell RNA-Seq and overview of cell quality control. (**A**) Procedures for collection, preparation and sequencing of ovine cumulus cell samples. (**B**) Doublet cells and probability distribution t-SNE plot. (**C**) Distribution of basic information about each cell sample before and after filtering. (**D**) Correlations between nFeature_RNA and nCount_RNA before and after filtering.

At 45 h after CIDR removal, the above 5 ewes selected from each *FecB* genotype group were anesthetized, a 5 cm long incision was created in the abdomen and the ovaries were exteriorized. Follicular fluid and cumulus–oocyte complex samples were aspirated from non-atretic follicles (diameter ≥ 3 mm) using a 22-gauge needle attached to a 2 ml sterile syringe containing 1 ml Dulbecco's phosphate-buffered saline (DPBS, Gibco, Grand Island, NY, USA) preheated to 37 °C^24^. Cumulus–oocyte complex samples were retrieved using a mouth pipette, and cumulus cell suspensions were obtained after hyaluronidase (10 mg/mL, Sigma, St Louis, MO, USA) treatment and DPBS re-suspension. The specific steps are shown in Fig. 1A.

### Cell counting, quality control, single-cell Gel Bead-In-EMulsion preparation and sequencing

The cumulus cell suspension was mixed with trypan blue solution (volume ratio of 9:1). Live and dead cells were evaluated under a microscope, and cell samples with > 90% viability were selected for single-cell Gel Bead-In-EMulsion (GEM) preparation and were barcoded based on the Chromium Next GEM Single Cell 3’ Reagent Kits v3.1 (10 × Genomics, Shanghai, China). Qualified cumulus cells from ewes of each *FecB* genotype were combined into one sample and diluted to 10^3^ ~ 10^4^ cells/μL. Then, these cells were barcoded and combined with primers and a master mix into GEMs. Full-length, barcoded cDNA was amplified via PCR; after enzyme digestion, the sequencing adapter P5 and primer R1 were added for the amplification of the sequencing library. Finally, the Illumina sequencing platform was used for the high-throughput sequencing of the constructed library.

### Establishment of single-cell gene expression matrix

The raw sequencing data were converted, filtered, aligned and quantified using the 10 × Genomics Cell Ranger software (Version 3.1.0) to produce a gene expression matrix for each recovered cell. Specifically, the raw BCL files were converted into FASTQ files; reads with low-quality barcodes and Unique Molecular Identifiers (UMIs) were filtered out; and the remaining genes were mapped to the sheep reference genome (https://www.ncbi.nlm.nih.gov/assembly/GCF_016772045.1/). Reads uniquely mapped to the transcriptome and intersecting an exon by at least 50% were considered for UMI counting. After correcting the UMI sequences, valid barcodes were identified based on the EmptyDrops method^25^. Cell-by-gene matrices were produced via UMI counting and cell barcode calling.

### Expression quality control and normalization

Cells detected with UMIs ≥ 8000 or mitochondrial gene percentages ≥ 10% and all cells with < 500 or > 4000 detected genes were excluded. Additionally, doublet cells were discarded based on the DoubletFinder tool (Version 2.0.3)^26^. After removing low-quality cells, the gene expression for each remaining cell was normalized using a global-scaling normalization method, “LogNormalize,” based on the following formula:

$$ {\text{Expression level of A gene in target cell }} = {\text{ log}}_{{\text{e}}} \left( {{1} + \frac{{\text{UMI A}}}{{\text{UMI Total}}} \times {10,000}} \right) $$

where UMI A represents the number of UMIs of the A gene in the target cell, and UMI Total represents the sum of all UMIs in the target cell.

### Cell clustering

Based on the Harmony algorithm, the cells were grouped by cell type rather than dataset-specific conditions so as to minimize batch effects and the influence of behavioral conditions on the clustering analysis^27^. Then, the normalized expression data were used in Principal Component Analysis (PCA) for dimensional reduction. Furthermore, the Seurat software was used to cluster and group the cells based on a graph-based clustering approach. The xpecific implementation steps included the following: based on the Euclidean distance in the PCA space, a shared nearest neighbor (SNN) graph was constructed and the edge weights between any two cells were refined based on Jaccard distance. Then, the Louvain method was applied to optimize the clustering. To better represent the classification results of various cell clusters, the t-distributed Stochastic Neighbor Embedding (t-SNE) method was used to achieve clearer separation between the clusters.

### Differentially expressed genes (up-regulation) and enrichment analysis

To characterize the transcriptional regulatory patterns and screen cell markers for individual cell clusters, the expression values of the detected genes in a given cluster were compared (Wilcoxon rank sum test) to those of the remaining cells^28^. Significantly upregulated genes were identified using the following criteria, First, the genes had to be at least 1.28-fold overexpressed in the target cluster. Second, the genes had to be expressed in > 25% of the cells belonging to the target cluster. Third, P < 0.01 was needed for significance. Using the phyper R package, Kyoto Encyclopedia of Genes and Genomes (KEGG) pathway enrichment analyses were conducted based on the identified upregulated, differentially expressed genes, to reveal the main features of each cluster^29^. The expression distributions of some important genes among the clusters or groups were visualized using bubble diagrams.

### Oxidative stress index evaluation in follicular fluid and cumulus cells

In our previous study, liquid chromatography–mass spectrometry and gas chromatography–mass spectrometry were adopted to detect the metabolic effects of the *FecB* gene in the follicular fluid and ovarian vein serum^14^. Briefly, samples (100 μL) were extracted through the automated MicroLab STAR® system (Hamilton Company, UT, USA) and centrifuged, and the resulting supernatants were analyzed via UPLC-MS/MS in positive and negative ion mode (UPLC: Waters, Milford, MA; mass spectrometer: Thermo-Finnigan LTQ, Thermo Fisher Scientific, Waltham, MA, scan range, 80–1000 m/z) and via GC–MS (Thermo-Finnigan Trace DSQ fast-scanning single-quadrupole mass spectrometer, scan range 50–750 m/z). The CVs for each intra- and inter-assay CV were < 10% and 15%, respectively^14,30^. Some metabolomic data from the follicular fluid of three *FecB* genotypes were used in this study to elucidate and validate the metabolic pathways screened via single-cell RNA-seq. In addition, follicular fluid and cumulus cells were collected from the preovulatory follicles to assess the oxidative stress indices. Using commercial assay kits purchased from the Nanjing Jiancheng Bioengineering Institute (Nanjing, China), the glutathione (GSH) content, superoxide dismutase (SOD) activity, total antioxidant capacity (T-AOC), malondialdehyde (MDA) content, and glutathione peroxidase (GSH-Px) activity were measured in accordance with the manufacturer's instructions. The GSH content (Kit No. A006-1) and SOD activity (Kit No. A001-1) of the follicular fluid and cumulus cells were measured using a microplate reader (Beijing Tianshi Tianli Medical Device Technology Development Center Co., Beijing, China) with absorbance of 420 nm and 550 nm, respectively. The T-AOC level (Kit No. A015-1), MDA content (Kit No. A003-1) and GSH-Px activity (Kit No. A005-1) of the follicular fluid and cumulus cells were determined using a spectrophotometer (Shanghai Jinghua Technology Instrument Co., Shanghai, China) with absorbance of 520 nm, 532 nm and 412 nm, respectively.

### Ethics approval and consent to participate

All experimental procedures mentioned in the present study were approved by the Science Research Department of the Chinese Academy of Science Animal Care and Use Committee (Approval 2022-147-106).

## Results

### Estrus phenotypes for ewes of three *FecB* genotypes under superovulation treatment

Under the superovulation treatment, the ewes of the three *FecB* genotypes showed no significant differences in the performance of the first estrus and last estrus and the estrus duration; meanwhile, the observed number of preovulatory follicles in the ovaries varied significantly across the three *FecB* genotype groups (Table 1). The concentrations of FSH, LH and P_4_ in the peripheral serum among the three *FecB* genotypes also showed no significant difference; however, there was a significant difference in the estradiol concentration, which was manifested in the fact that the BB and WB ewes with *FecB* mutations had significantly lower values than the wild-type WW individuals (Table 2).

**Table 1:** Estrus and preovulatory follicle number in ewes with three *FecB* genotypes under superovulation treatment.

Genotype (N)	First estrus (h)	Last estrus (h)	Estrus duration (h)	Preovulatory follicles number
WW (8)	10.67 ± 1.67	41.33 ± 1.56	30.67 ± 1.56	19.56 ± 2.23^c^
WB (8)	12.00 ± 2.00	40.67 ± 1.94	28.67 ± 3.13	41.56 ± 4.07^a^
BB (8)	13.80 ± 1.56	40.80 ± 1.50	27.00 ± 2.24	37.10 ± 3.13^b^

In the same column, values with different lowercase superscript letters indicate significant differences (*P* < 0.05), while the same superscript letter or no superscript letter indicates no significant difference (*P* > 0.05).

**Table 2:** Peripheral serum concentrations of FSH, LH, P_4_ and E_2_ in ewes with three *FecB* genotypes under superovulation treatment.

Genotype (N)	FSH (mIU/mL)	LH (mIU/mL)	P_4_ (ng/mL)	E_2_ (pg/mL)
WW (5)	3.71 ± 0.33	6.18 ± 1.01	0.27 ± 0.06	38.14 ± 5.16^a^
WB (5)	3.56 ± 0.29	6.56 ± 0.86	0.30 ± 0.01	22.78 ± 2.67^b^
BB (5)	3.54 ± 0.35	7.70 ± 1.22	0.23 ± 0.04	18.62 ± 2.07^b^

In the same column, values with different lowercase superscript letters indicate significant differences (*P* < 0.05), the same superscript letter or no superscript letter indicates no significant difference (*P* > 0.05).

### Overview of single-cell RNA-Seq data for cumulus cells in ovine graafian follicles

The three *FecB* genotypes of cumulus cells (BB, WB and WW) were isolated from the follicles (≥ 3 mm) 45 h after CIDR removal (Fig. 1A). For each genotype, the cumulus cells from five ewes were combined. All samples were then prepared for single-cell RNA-seq based on the 10 × Genomics platform. More than 10,000 cells in each group were captured and analyzed (detailed information identified by CellRanger is provided in Supplementary File S1). After quality control, valid barcodes were > 96.90%, and valid UMIs were > 99.9%, whereas the Q30 bases in barcodes for each group were > 95.10%. The fraction reads in the cells were > 77.60%, and ≥ 85.10% of the filtered reads were mapped to the genome for all samples. At this point, the reads were annotated as specific genes, and the median number of genes per cell detected in the above three samples ranged from 1529 to 2414 after the analysis of the gene expression data from CellRanger and Seurat quality control. Further, doublet and abnormal cells were excluded using CellRanger strategies, with 10,289, 9,378 and 8,873 cells from BB, WB and WW obtained for subsequent analyses (Fig. 1B,C, Supplementary File S2). The correlations between the gene number/cell (nFeature_RNA) and UMI count (nCount_RNA) before and after filtering the cells were 0.89 and 0.93, respectively (Fig. 1D), confirming the successful library construction for scRNA-seq.

### *FecB* mutation affected cell clustering for cumulus cells in ovine graafian follicles

**Figure 2:**
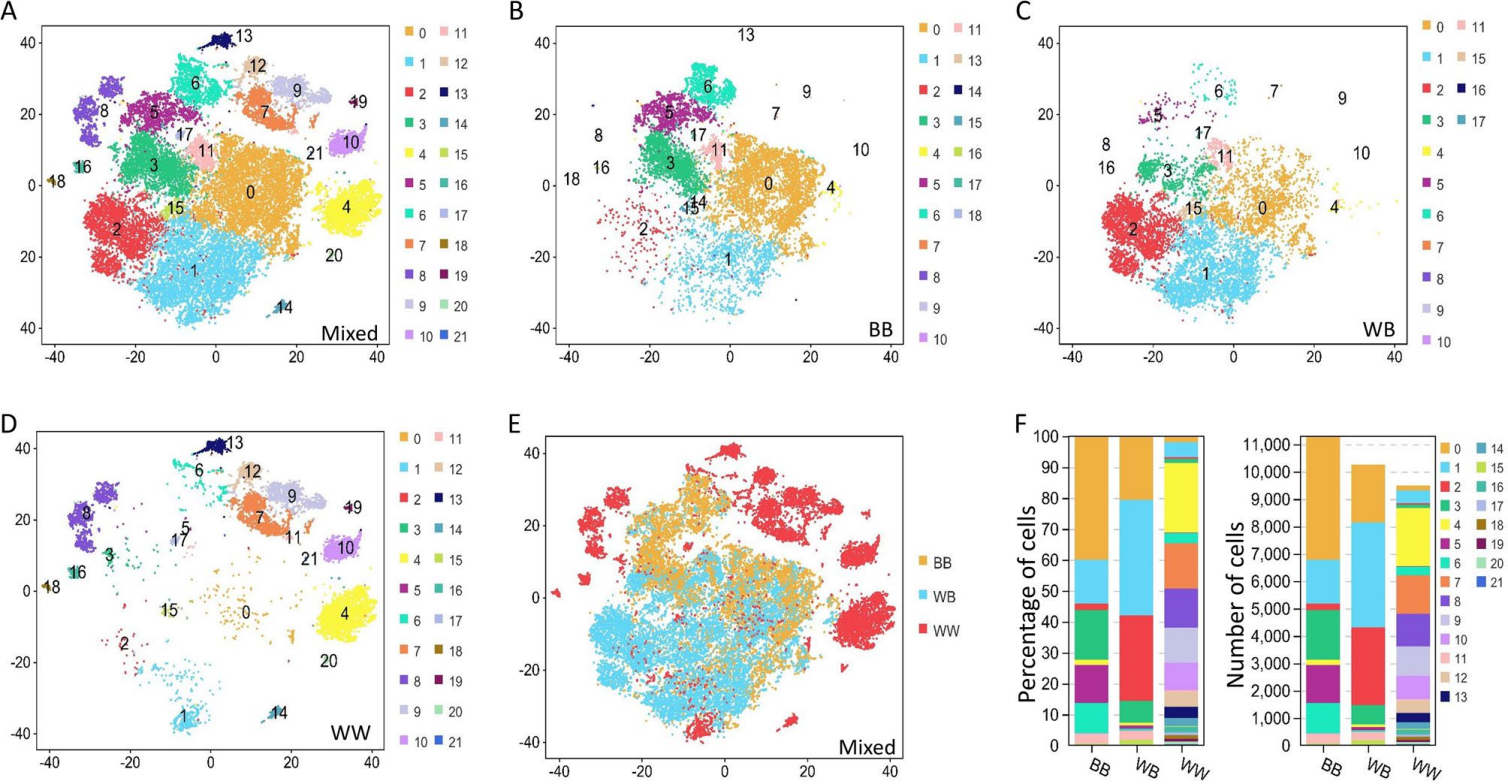
Cell clustering of cumulus cells in ovine Graafian follicle. (**A**) Cell clustering of cumulus cells for all three *FecB* genotypes. (**B**) Cell clustering of cumulus cells for BB genotype. (**C**) Cell clustering of cumulus cells for WB genotype. (**D**) Cell clustering of cumulus cells for WW genotype. (**E**) Distribution of three *FecB* genotypes of cumulus cells in t-SNE plot. (**F**) Stacked plot of cell numbers and percentages for 22 cell clusters in three *FecB* genotype samples.

Cells with similar expression patterns were grouped together using a nonlinear clustering method and the results were visualized using the t-Distributed Stochastic Neighbor Embedding (t-SNE) diagram; for the three *FecB* genotypes, 22 cell clusters were detected in the cumulus cells (Fig. 2A). Only 18 and 15 cell clusters were detected in the BB and WB groups (Fig. 2B,C), whereas all 22 cell clusters were detected in WW ewes (Supplementary File S3, Fig. 2D). The distributions of the three *FecB* genotypes of cumulus cells are provided in the t-SNE diagram (Fig. 2E). Cell clusters 0, 1, 2 and 3 together accounted for 75.25% and 92.63% of the total cell counts in the BB and WB groups, but these four cell clusters accounted for only 8.63% in the WW group (Fig. 2F).

### Annotation of immune cells and cumulus cells in ovine graafian follicles

**Figure 3:**
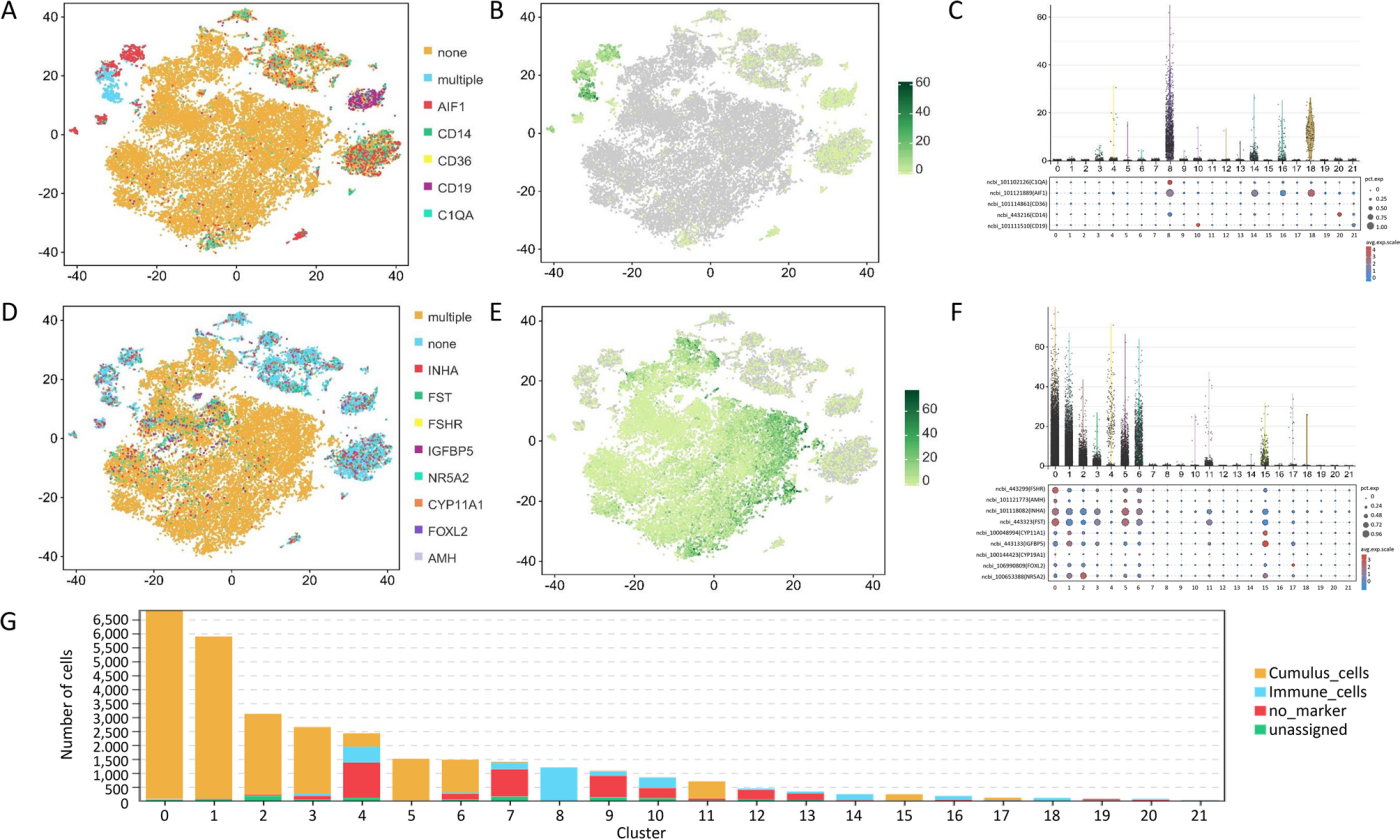
Annotation of immune cells and cumulus cells in ovine preovulatory follicles. (**A**) Immune cells were annotated with the following marker genes: AIF1, CD14, CD36, Cd19 and C1QA. (**B**) Aggregate expression abundance of marker genes for all immune cells. (**C**) Violin and bubble plots for expression of immune cell marker genes. (**D**) Cumulus cells annotation with marker genes (*INHA*, *FST*, *FSHR*, *IGFBP5*, *NR5A2*, *CYP11A1*, *FOXL2* and *AMH*). (**E**) Aggregate expression abundance of marker genes for all cumulus cells. (**F**) Violin and bubble plots for expression of cumulus cell marker genes. (**G**) Stacked plot of cell numbers for annotated cells in all 22 cell clusters.

Under the in vivo sampling conditions, the abundant microvessels around the mature follicles were expected to rupture, with many blood-derived immune cells contaminating the aspirated follicular fluid. To ensure the purity of the cumulus cells in subsequent analysis, immune cells were distinguished and filtered based on relevant cell markers (AIF1, CD14, CD36, Cd19 and C1QA; Fig. 3A). The UMI counts (expression abundance) of these five immune cell markers were relatively low in all three groups of collected cells (Fig. 3B). Based on the violin and bubble plots of the immune cell marker gene expression, cell clusters 4, 8, 14, 16 and 18 were classified as immune cells and attributed to ruptured microvessels (Fig. 3C).

Combining a cell marker database (http://xteam.xbio.top/CellMarker/index.jsp) and the published literature^31,32^, *INHA*, *FST*, *FSHR*, *IGFBP5*, *NR5A2*, *CYP11A1*, *FOXL2* and *AMH* were used as marker genes for cumulus cells (Fig. 3D). From Fig. 3E, we can conclude that cumulus cells comprised the majority of the collected cells in the present experiment (Fig. 3E). Furthermore, the cumulus cells were mainly distributed in cell clusters 0, 1, 2, 3, 4, 5, 6, 11, 15 and 17 (Fig. 3F,G, Supplementary File S4).

### Re-clustering analysis of ovine cumulus cells in graafian follicles

**Figure 4:**
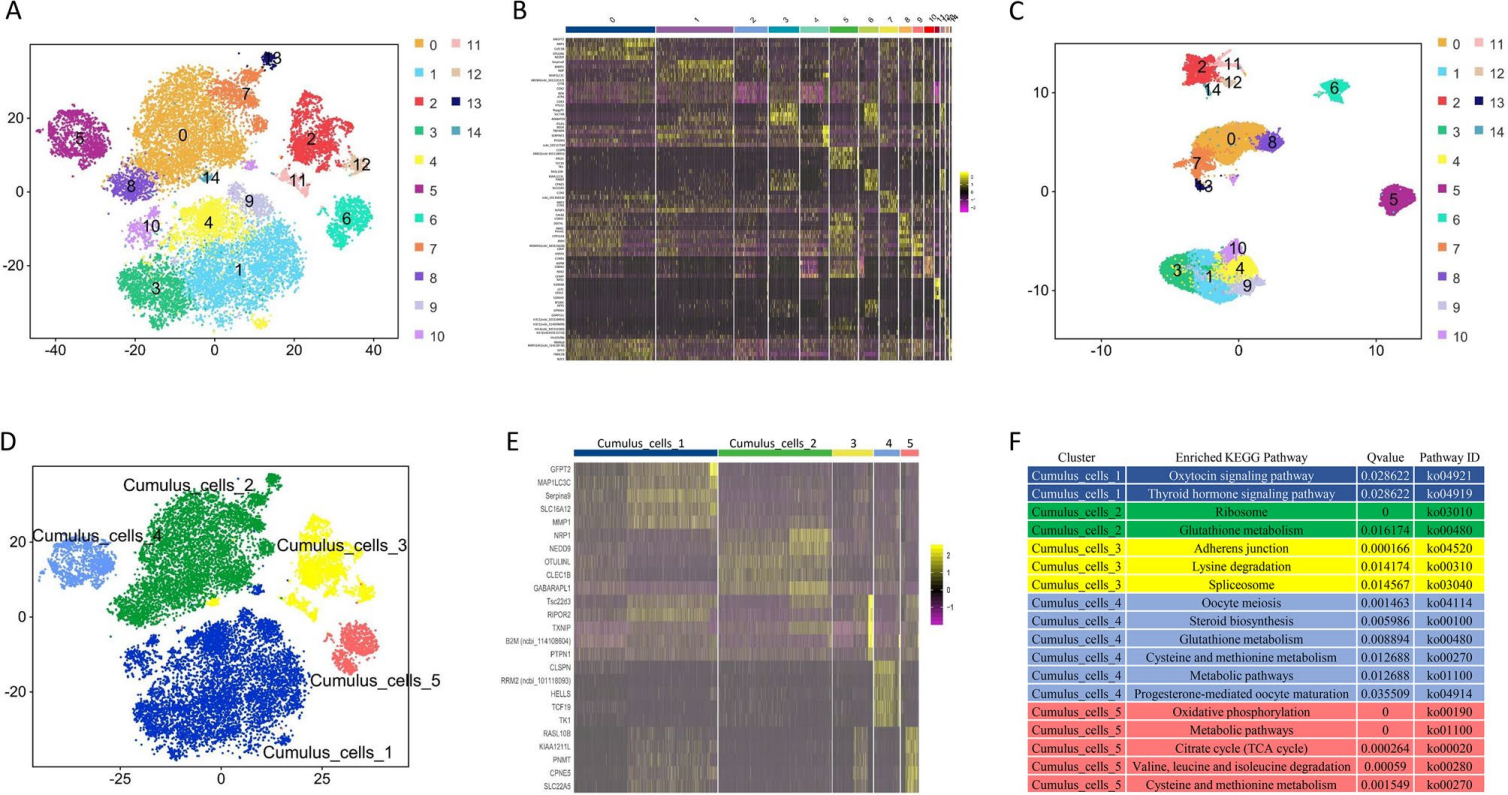
Re-clustering analysis of ovine cumulus cells in preovulatory follicles. (**A**) Re-clustering analysis of annotated cumulus cells. (**B**) Screening of marker genes in 15 clusters of cumulus cells using heat map analysis. (**C**) Further clustering analysis of 15 cumulus cell clusters based on UMAP map. (**D**) Distribution of cumulus_cells_1-5 in t-SNE plot. (**E**) Screening of marker genes in Cumulus_cells_1-5 using heat map analyses. (**F**) KEGG enrichment analysis of genes with significant, differentially upregulated expression for each group of cumulus cells.

Based on the above results, the cumulus cells, i.e., cell clusters 0, 1, 2, 3, 4, 5, 6, 11, 15 and 17, were the focus of the subsequent analyses. According to gene expression characteristics, these 10 cell clusters were subjected to a re-clustering analysis, and the cumulus cells were re-divided into 15 cell clusters (Fig. 4A). As shown in Fig. 4B, each of the 15 clusters of cells had its own characteristic expression gene (Supplementary File S5). Using UMAP analysis, these 15 cluster cells were further divided into five clusters of cumulus cells (Fig. 4C). Cell clusters 1, 3, 4, 9 and 10 were defined as Cumulus_cells_1; cell clusters 0, 7, 8 and 13 were defined as Cumulus_cells_2; cell clusters 2, 11, 12 and 14 were defined as Cumulus_cells_3; cell cluster 5 were defined as Cumulus_cells_4; and cell cluster 6 were defined as Cumulus_cells_5 (Fig. 4D).

Marker genes of Cumulus_cells_1-5 were screened out using the gene expression heat map (Fig. 4E, Supplementary File S6). Furthermore, genes exhibiting significant, differentially upregulated expression for each cumulus cell were selected for KEGG enrichment analysis. These genes with upregulated expression in Cumulus_cells_1 were mainly enriched in the oxytocin signaling pathway and thyroid hormone signaling pathway; genes with upregulated expression in Cumulus_cells_2 were mainly enriched in the ribosome and glutathione metabolism pathway; genes with upregulated expression in Cumulus_cells_3 were mainly enriched in adherens junction, lysine degradation and spliceosome pathway; genes with upregulated expression in Cumulus_cells_4 were mainly enriched in oocyte meiosis, steroid biosynthesis, glutathione metabolism, cysteine and methionine metabolism, metabolic pathways and progesterone-mediated oocyte maturation; and genes with upregulated expression in Cumulus_cells_5 were mainly enriched in oxidative phosphorylation, metabolic pathways, TCA cycle and other amino acid pathways (Fig. 4F).

### Pseudo-time trajectories of ovine cumulus cells

**Figure 5:**
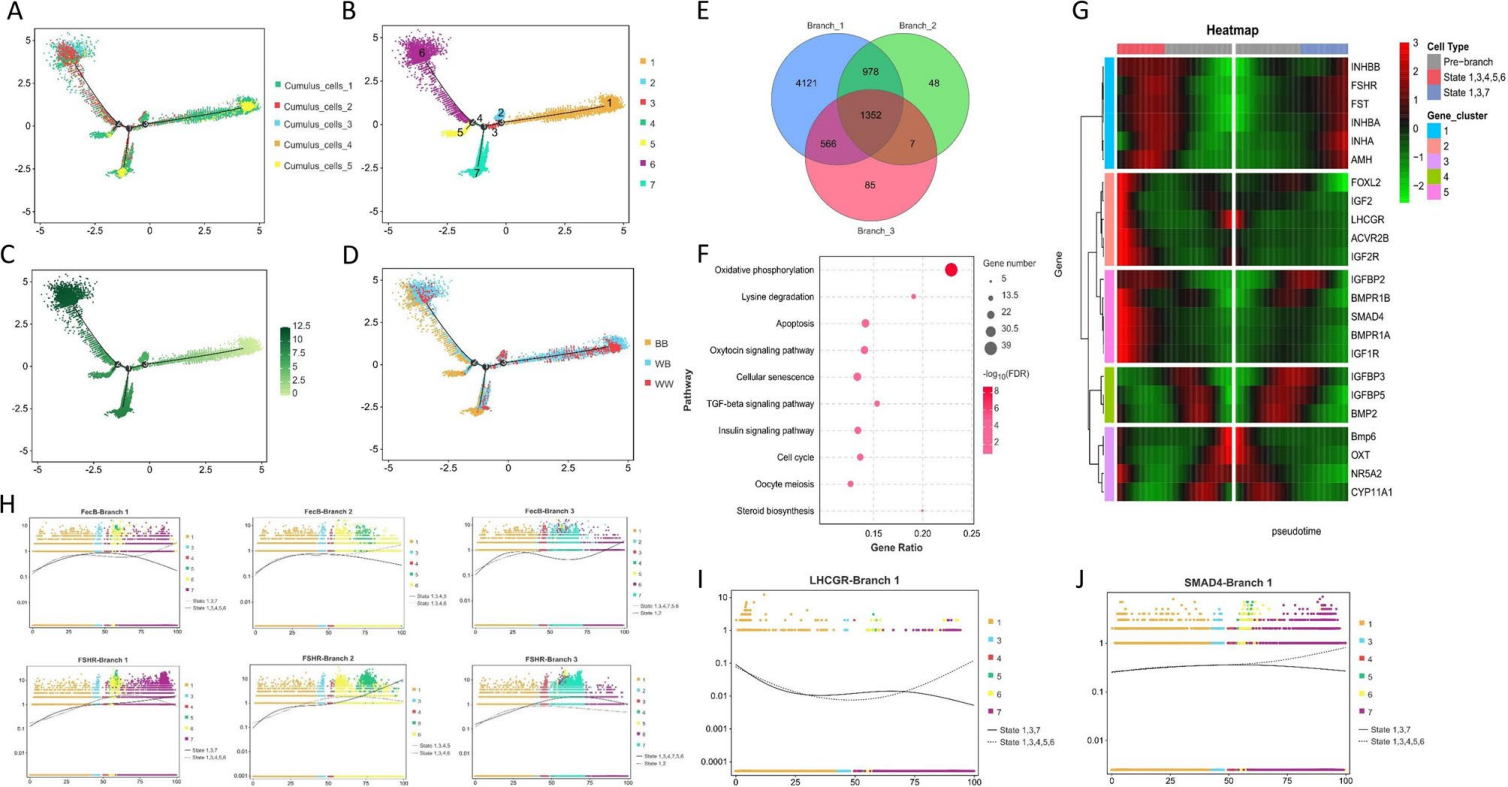
Pseudo-time trajectories of ovine cumulus cells in preovulatory follicles. (**A**) Pseudo-time trajectories of Cumulus_cells_1-5. (**B**) Seven cell states formed in pseudo-time trajectory analysis. (**C**) Differentiation stages for all cumulus cells. (**D**) Distribution of cumulus cells of three *FecB* genotypes in various differentiation stages. (**E**) Venn diagram for differentially expressed genes of three branch nodes. (**F**) KEGG enrichment analysis of 1,352 common differentially expressed genes. (**G**) Differentially expressed genes of differentiation fate displayed as heat maps for branch node 1. (**H**) Expression of *FecB* and *FSHR* genes in all three branch nodes. (**I**) *LHCGR* gene expression in branch node 1. (**J**) *SMAD4* gene expression in branch node 1.

Pseudo-time trajectory analyses of Cumulus_cells_1-5 can help to predict changes in and the differentiation of cumulus cells throughout the timeline. Cumulus_cells_1-5 underwent three cell differentiation branch nodes and formed seven cell states (Fig. 5A,B). Cell state 1 in Fig. 5C, labeled with a lighter color, represents cumulus cells at the earlier stages of follicle development, whereas darker-labeled cell states represent cumulus cells at the later stages of follicle development. These cumulus cells, from all three *FecB* genotypes, were present in all seven cell states. Compared to those of the WB and WW genotypes, BB cumulus cells appeared more differentiated (later stage) on the pseudo-time axis (Fig. 5D).

The differentially expressed genes of the three branch nodes were analyzed and displayed in Venn diagrams. There were 1,352 differentially expressed genes in common among the three branch nodes (Fig. 5E), and the number of differentially expressed genes in branch node 1 occupied the absolute dominant position. Based on the KEGG enrichment analysis, these 1,352 differentially expressed genes were mainly enriched in oxidative phosphorylation, the oxytocin signaling pathway, the TGF-beta signaling pathway, and oocyte meiosis (Fig. 5F).

For the main branch node 1, the differentially expressed genes with differentiation fates were displayed via heat maps. The expression of BMP/SMAD signaling pathway and reproductive hormone response pathway genes, including *FecB* (*BMPR1B*), had important roles in the differentiation fates of the cumulus cells (Fig. 5G, Supplementary File S7). Candidate genes such as *FecB* and *FSHR* were differentially expressed in all three branch nodes (Fig. 5H), whereas the *LHCGR* and *SMAD4* genes were only differentially expressed in branch node 1 (Fig. 5I,J).

### Differential expression and enrichment analysis of cumulus cells genes

**Figure 6:**
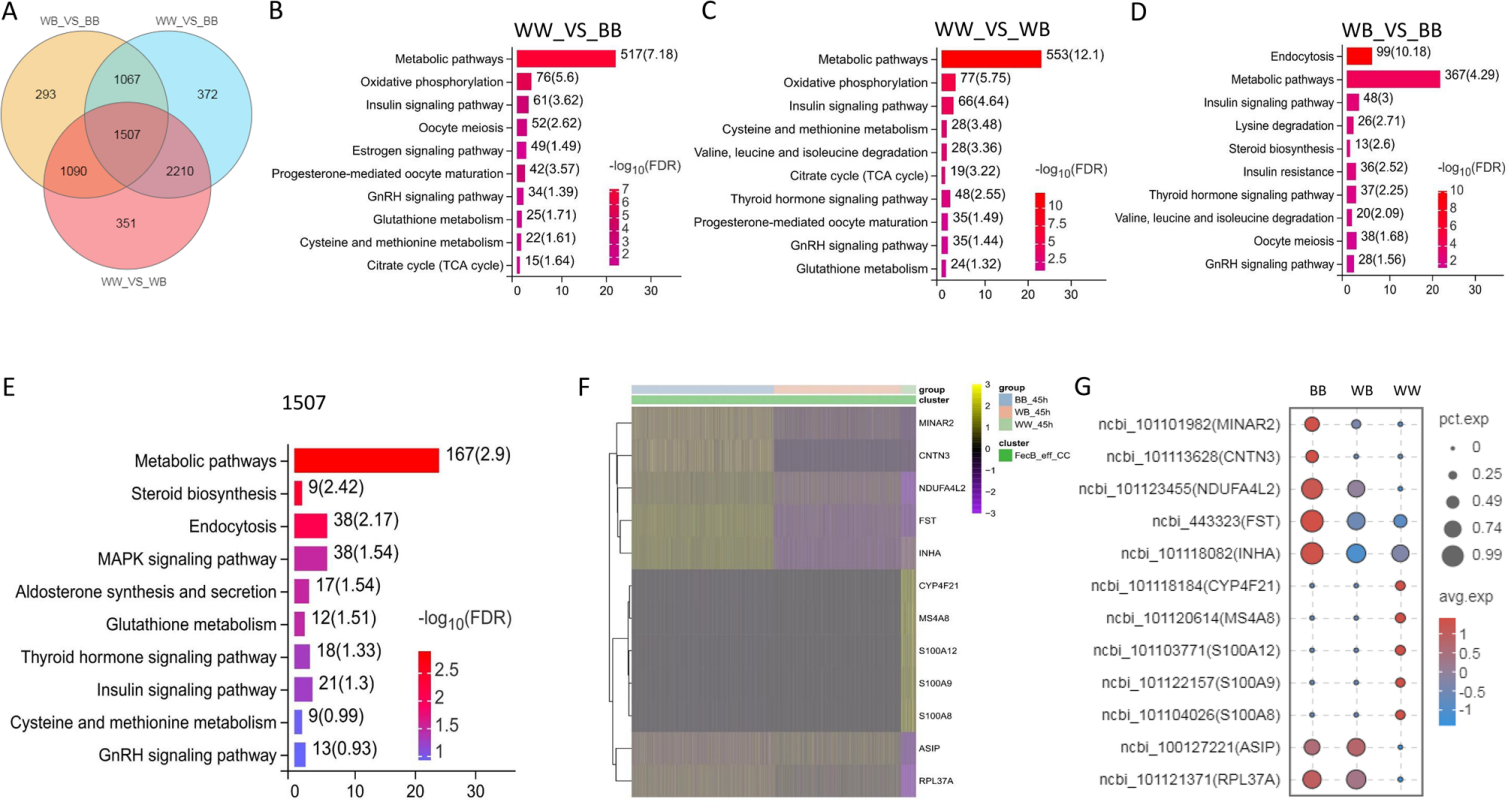
Differentially expressed genes and their enrichment analysis in cumulus cells of three *FecB* genotypes. (**A**) Venn diagram for differentially expressed genes of each pairwise comparison. (**B**) KEGG enrichment analysis of differentially expressed genes for WW_VS_BB. (**C**) KEGG enrichment analysis of differentially expressed genes for WW_VS_WB. (**D**) KEGG enrichment analysis of differentially expressed genes for WB_VS_BB. (**E**) KEGG enrichment analysis of 1,507 common differentially expressed genes. (**F**) Screening of marker genes with *FecB* genotypes in cumulus cells using heat map analysis. (**G**) Screening of marker genes with *FecB* genotypes in cumulus cells using bubble chart analysis.

For the cumulus cells of the three *FecB* genotypes, significant differentially expressed genes in each pairwise comparison were identified using Venn diagram analysis (Fig. 6A). In total, 1,507 common differentially expressed genes were identified among the pairwise comparisons of the three *FecB* genotypes. The pairwise comparisons of BB and WB yielded the lowest number of differentially expressed genes; however, each was accompanied by more differentially expressed genes when compared to the WW genotype. Among them, the WW_VS_BB and WW_VS_WB differentially expressed genes were mainly enriched in signaling pathways such as the metabolic pathway, oxidative phosphorylation, glutathione metabolism and the TCA cycle (Fig. 6B,C). Meanwhile, the WB_VS_BB differentially expressed genes were mainly enriched in signaling pathways such as endocytosis, the metabolic pathway and the insulin signaling pathway; they were not enriched in signaling pathways such as oxidative phosphorylation, glutathione metabolism and the TCA cycle (Fig. 6D). Regarding the 1,507 common differentially expressed genes, based on the KEGG enrichment analysis, these genes were mainly enriched in metabolic pathways, glutathione metabolism, cysteine and methionine metabolism (Fig. 6E). Based on the heat map and bubble chart (Fig. 6F,G, Supplementary File S8), *MINAR2*, *CNTN3*, *NDUFA4L2*, *FST*, *INHA* and other genes were significantly highly expressed in the BB group; genes *CYP4F21*, *MS4A8*, *S100A12*, *S100A9* and *S100A8* were significantly highly expressed in the WW group; and *ASIP* and *RPL37A* were significantly lowly expressed in the WW group. These differentially expressed genes have potential as marker genes used to identify the *FecB* genotype in cumulus cells.

### Analysis of oxidative stress and related metabolic intermediates in graafian Follicles

**Figure 7:**
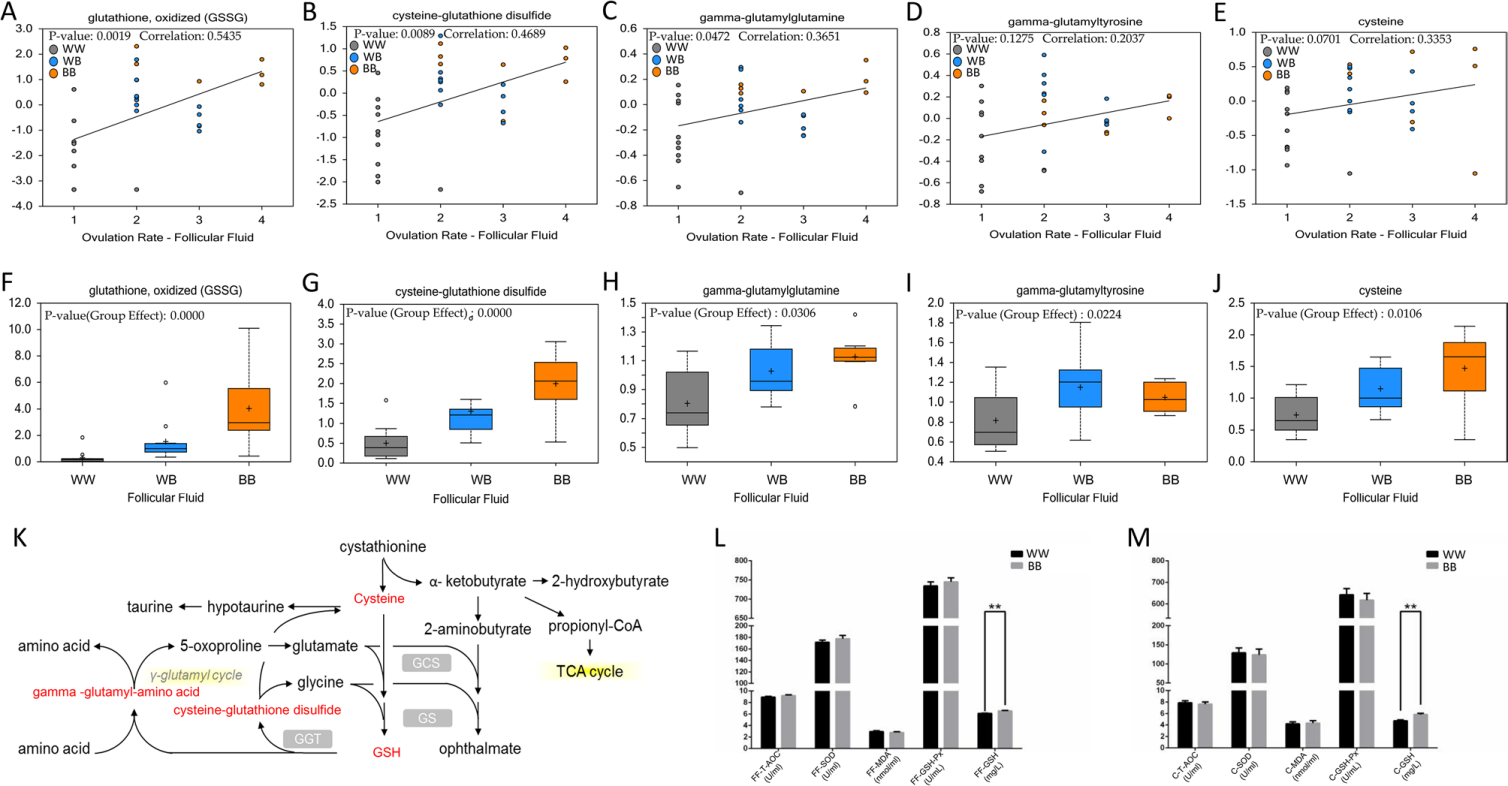
Effects of *FecB* mutations on oxidative stress and related metabolic intermediates in preovulatory follicles. (**A**) Correlation between concentrations of GSSG in follicular fluid and ovulation rate. (**B**) Correlation between follicular fluid concentrations of cysteine-glutathione disulfide and ovulation rate. (**C**) Correlation between follicular fluid concentrations of gamma-glutamylglutamine and ovulation rate. (**D**) Correlation between follicular fluid concentrations of gamma-glutamyltyrosine and ovulation rate. (**E**) Correlation between follicular fluid concentrations of cysteine and ovulation rate. (**F**) GSSG concentration distribution of three *FecB* genotypes in follicular fluid. (**G**) Cysteine-glutathione disulfide concentration distribution of three *FecB* genotypes in follicular fluid. (**H**) Gamma-glutamylglutamine concentration distribution of three *FecB* genotypes in follicular fluid. (**I**) Gamma-glutamyltyrosine concentration distribution of three *FecB* genotypes in follicular fluid. (**J**) Cysteine concentration distribution of three *FecB* genotypes in follicular fluid. (**K**) GSH anabolic pathway map; red font indicates that concentration of metabolic intermediates was significantly elevated in BB. (**L**) Indicators of oxidative stress in follicular fluid for BB and WW genotypes. (**M**) Indicators of oxidative stress in cumulus cells for BB and WW genotypes.

Liquid chromatography mass spectrometry and gas chromatography–mass spectrometry were used to explore the oxidative stress and related metabolic intermediates in the ovarian follicular fluid of the three *FecB* genotype groups. Spearman correlation analyses were used to determine the correlations of oxidized glutathione, cysteine-glutathione disulfide, gamma-glutamylglutamine, gamma-glutamyltyrosine and cysteine with the ovulation rate. The above-mentioned metabolic intermediates involved in glutathione metabolism were positively correlated with the ovulation rate in the sheep (Fig. 7A–K). In the further analyses, the levels of GSH in the follicular fluid and cumulus cells of the BB genotype were significantly higher than those of the WW genotype (Fig. 7L,M). This corroborated the results of the single-cell RNA sequencing and implied that the follicular fluid and cumulus cells of the high-fecundity BB group had a higher antioxidant stress capacity than those of the WW group.

## Discussion

As a valid molecular marker for the ovulation rate and litter size in sheep, the *FecB* gene mutation has been widely used in sheep breeding in China, India, Israel and other countries^33^. In China, the breeding of Luxi Black Head Sheep, Luzhong mutton sheep and Huang-huai sheep involves *FecB* gene molecular marker-assisted selection^34–36^. However, the molecular mechanism by which *FecB* gene mutations regulate the ovulation rate in sheep is uncertain. To date, differences among sheep with various *FecB* genotypes have been studied based on reproductive hormones, the expression of gonadal axis genes, follicular fluid metabolomics and in vitro cell cultures^10,11,14,37,38^. In this study, we found that the number of preovulatory follicles formed in the three *FecB* genotypes of ewes treated with FSH was significantly different, and its trend was consistent with that of ewes in a natural estrus. This suggests that the *FecB* mutation affects the ovarian response to FSH. Therefore, the *FecB* genotype, when detected in advance, may be helpful in predicting the number of ovulations and embryos obtained after superovulation treatment. In addition, the first estrus and last estrus for the three *FecB* genotypes of ewes in the present study were significantly advanced compared with those in non-superovulation ewes, but the estrus duration did not change^13^. In the peripheral blood serum hormone comparison, we speculated that the number of follicles formed in the +  + ovaries was smaller and the volume was larger than that in *FecB* mutation carriers, which resulted in a higher concentration of estradiol secreted by the follicular membrane.

The 10 × single-cell transcriptome technique provided a novel method to investigate the effects of the *FecB* gene mutation on cumulus cell clustering and differentiation. In the present study, the expression patterns of the cumulus cells differed between the *FecB* carriers and wild-type ewes. This was similar to a previous metabolomics study on the effects of the *FecB* gene mutation on the composition of the follicular fluid in sheep—namely, there was a large gap between individuals with the *FecB* mutation and non-carriers^14^, supporting the hypothesis that the cumulus cell expression patterns are affected by the *FecB* mutation. In a recent 10 × Genomics single-cell RNA-seq study on the ovarian somatic cells of Hu sheep, there were expression differences regarding a high and low litter size in the ovary, which were mainly reflected in granulosa cells^39^. In the present study, we further isolated cumulus cells from the Graafian follicles as subjects for single-cell transcriptome analysis, which allowed us to clearly elucidate the transcriptional regulatory effects of the *FecB* gene mutation in cumulus cells. To achieve this goal, and based on previous work on follicle synchronization and superstimulation^40^, we generated preovulatory follicles, aspirated the follicular fluid and rigorously acquired cumulus cells via mouth pipetting and hyaluronidase in vitro. Nonetheless, in the process of single-cell sampling and separation, other cell types remained mixed with the target cells. To obtain a purer single-cell sample for the subsequent analysis, appropriate target cell markers were selected to assist in determining the source of the sampled cells. The cell markers used to identify the cumulus and immune cells in this study were mainly obtained from the CellMarker database, as well as other reports^39,41,42^. Heat map analyses were used to screen new cell markers for each cell cluster. There were significant differences in the expression of FST and INHA genes in the cumulus cells of the three *FecB* genotypes, implying that FST and INHA may be acceptable as granulosa cell markers, although the markers of cumulus cells remain to be determined.

Using sampling and cell marker screening, the cumulus cells were further divided into distinct cell clusters. Based on the pseudo-time trajectory analyses, the differentiation states and fates of these screened cumulus cells differed. Heterogeneity in gene expression is prevalent among single cells^43^. The high expression of LHCGR in cumulus cells implies that the preovulatory follicle was ready to ovulate in response to an LH surge^44^. In this study, more cumulus cells were concentrated in cell state 6, in the late stage of differentiation, and it was observed that the expression of *LHCGR* and *SMAD4* in this state gradually increased, whereas the expression of *FSHR* and *FecB* gradually decreased. Similarly, follicles that originally relied on FSH signaling for growth gradually switched to LH signaling^45^. *SMAD4* is the downstream gene of the *FecB* (*BMPR1B*) gene, and they both belong to the TGF-β signaling pathway^46^. In this study, the high expression of the *SMAD4* gene and the low expression of the *FecB* gene in cell state 6 indicated that the differentiation of cumulus cells tends to occur late at this stage. This inference is supported by a report indicating that, as the terminal regulatory molecule in TGF-β signaling, *SMAD4* can be identified as an anti-apoptosis factor in cumulus cells^47^.

When the differentially expressed genes of the three *FecB* genotypes in the cumulus cells were analyzed, the number of differentially expressed genes between the BB and WB genotypes was relatively small, whereas there were more differentially expressed genes between *FecB* carriers and wild types. This was consistent with the results of the cell clustering of the three *FecB* genotypes of cumulus cells in the present study and with the phenotypic effects of the ovulation rate and litter size with *FecB* gene mutations. The common differentially expressed genes of the three *FecB* genotypes were enriched in metabolic pathways, glutathione metabolism amd cysteine and methionine metabolism, corroborated by our previous results regarding the follicular fluid metabolomics of the three *FecB* genotypes^14^. The oxidative phosphorylation pathway was significantly enriched in the differentially expressed genes among the cell differentiation branch nodes of the cumulus cells, as well a in the differentially expressed genes in the cumulus cells among the three *FecB* genotypes. Therefore, studying the oxidative phosphorylation of cumulus cells should provide insights into the molecular mechanisms affecting the differentiation of cumulus cells and the effects of the *FecB* gene. Cumulus cells metabolize 23-fold more glucose (per ml tissue/h) than oocytes and oocytes are almost entirely reliant on cumulus cells for the uptake and supply of glucose and its metabolites, either via gap junctions or from the surrounding fluid following cumulus cell secretion^48,49^. During oocyte maturation, cumulus cells provide the oocytes with substrates for the TCA cycle and oxidative phosphorylation^50^, consistent with the significant enrichment of the oxidative phosphorylation pathway in our study.

As oocytes use oxygen to produce energy through mitochondrial oxidative phosphorylation, oocytes coated with cumulus cells are the main source of ROS^51^. Diplotene-arrested oocytes morphologically identified by a germinal vesicle (GV) were triggered by ROS to resume meiosis within preovulatory follicles^52^. The increased accumulation of ROS in cumulus–oocyte complexes could lead to oxidative stress, which directly or indirectly reduces oocytes’ quality by inducing the apoptosis of cumulus cells and oocytes^53,54^. Follicular fluid and cumulus cells contain superoxide dismutase (SOD), glutathione peroxidase (GSH-Px) and other antioxidant molecules that protect oocytes from oxidative damage and maintain the redox status during the final stages of folliculogenesis^55^. Based on the various oxidative stress indicators in the cumulus cells and follicular fluid of the mutant *FecB* genotype considered in the present study, combined with previous metabolomics studies, we suggest that *FecB* gene carriers have larger potential antioxidant reserves in their pre-ovulation Graafian follicles than wild-type individuals. Consequently, the ovaries of ewes with the *FecB* gene can better manage ROS homeostasis.

## Conclusions

In summary, *FecB* mutations can cause differences in ewes’ responses to exogenous reproductive hormones. The cumulus cell expression patterns differed between *FecB* carriers and wild-type individuals. The screened cumulus cells could be further divided into cell clusters. The differentiation states and fates of each group of cumulus cells remained different, supporting the notion that the phenomenon of heterogeneity in gene expression in single cells is prevalent. The oxidative phosphorylation pathway was significantly enriched in the differentially expressed genes among the cell differentiation branch nodes of cumulus cells, as well as in the differentially expressed genes of cumulus cells among the three *FecB* genotypes. Combined with the important role of oxidative phosphorylation in the maturation of COCs, we speculate that the study of the oxidative phosphorylation of cumulus cells will yield new knowledge regarding the molecular mechanisms of cumulus cell differentiation and the mutation effect of the *FecB* gene.

### Supplementary Information

Supplementary Information.

## Data Availability

The raw data have been deposited at China National Center for Bioinformation, the BioProject ID (PRJCA021455) and Biosample accessions (SAMC3180445, SAMC3180444, SAMC3180443) are publicly available.

## Abbreviations

STH: Small Tail Han
*FecB*: Fecundity Booroola
*BMPR1B*: Bone morphogenetic protein receptor type-1B
FSH: Follicle-stimulating hormone
PMSG: Pregnant mare serum gonadotropin
LH: Luteinizing hormone
E_2_: Estradiol
P_4_: Progesterone
PCA: Principal component analysis
COCs: Cumulus-oocyte complexes
CIDR: Controlled internal drug releasing
GSH: Glutathione
SOD: Superoxide dismutase
T-AOC: Total antioxidant capacity
MDA: Malondialdehyde
GSH-Px: Glutathione peroxidase
KEGG: Kyoto encyclopedia of genes and genomes
